# Magnetic, Optical and Phonon Properties of Ion-Doped MgO Nanoparticles. Application for Magnetic Hyperthermia

**DOI:** 10.3390/ma16062353

**Published:** 2023-03-15

**Authors:** Iliana Apostolova, Angel Apostolov, Julia Wesselinowa

**Affiliations:** 1 University of Forestry, Kl. Ohridsky Blvd. 10, 1756 Sofia, Bulgaria; 2 University of Architecture, Civil Engineering and Geodesy, Hristo Smirnenski Blvd. 1, 1046 Sofia, Bulgaria; 3 Faculty of Physics, Sofia University “St. Kliment Ohridski”, J. Bouchier Blvd. 5, 1164 Sofia, Bulgaria

**Keywords:** MgO nanoparticles, ion doping, room-temperature ferromagnetism, magnetic hyperthermia, band gap, phonon energy

## Abstract

The influence of size and doping effects on the magnetization *M*, phonon ω and band gap energy $E_{g}$ of MgO nanoparticles is studied using a microscopic model. The room-temperature ferromagnetism is due to surface or/and doping effects in MgO nanoparticles (NPs). The influence of the spin–phonon interaction is discussed. *M* increases with decreasing NP size. *M* and $E_{g}$ can increase or decrease by different ion doping (Co, Al, La, Fe) due to the different strain that appears. It changes the lattice parameters and the exchange interaction constants. We found that MgO NP with size of 20 nm and Fe- or Co-doping concentration *x* = 0.1 and *x* = 0.2, respectively, have a Curie temperature $T_{C}$ = 315 K, i.e., they are appropriate for application in magnetic hyperthermia, they satisfy the conditions for that. The energy of the phonon mode ω = 448 cm${}^{-1}$ increases with decreasing NP size. It increases with increasing Co and Fe, or decreases with Sr ion doping.

## 1. Introduction

MgO nanoparticles (NPs) have been intensively studied in recent years due to their special electronic and magnetic properties [1,2,3,4]. MgO NPs are used in a variety of industrial applications. Recently, they have found application in the biomedical field for cancer therapy [2]. Many researchers note that bulk MgO is nonmagnetic, it is diamagnetic, while its NP structure confirms room-temperature ferromagnetism (RTFM) [5,6,7]. Doping is an important tool for the modification of the physical properties of metal oxide NPs. The saturation magnetism was found to be changed due to ion doping. The RTFM of MgO NPs doped with different ions, such as Pb, Zn, Mn, Ti, Cr, Fe, Ni, Al, Ag, Eu, La, etc., are observed experimentally by many authors [8,9,10,11,12,13]. The reported weak RTFM is mainly due to oxygen or Mg vacancies on the surface [7,10,14]. Kumar et al. [15] and Li et al. [16] have shown that possibly the existence of oxygen vacancies and adsorbed H${}^{-}$ species are important for the appearance of RTFM in MgO nanocrystals or thin films, respectively. Ion doping also influences the optical and electro-optical properties of MgO NPs [17,18,19,20,21,22].

Ab initio analysis was used by Obeid et al. [23] to evaluate the magnetic and elastic properties of Co-doped MgO NPs. The phonon densities of states in dependence on the lattice parameters of MgO were calculated by Wang et al. [24] using the phonon theory. Recently, the Hartree–Fock wave functions of MgO clusters have been determined [25].

Raman scattering lines of MgO were observed by Schlecht et al. [26]. Very good agreement was obtained between the observed lines and the lattice dynamic theory of finite crystals, whereas no agreement was found with the macroscopic theory. Ab initio studies within the local-density approximation of phonons including LO mode in MgO were performed by Lazewski et al. [27]. The authors calculated the Hellmann–Feynman forces for the cubic and elongated supercells of the MgO crystal. The anharmonic lattice dynamics of MgO have been studied at high temperatures by infrared spectroscopy and inelastic X-ray scattering measurements combined with density functional perturbation theory calculations by Guira et al. [28]. Chen et al. [29] studied by the density functional theory of Raman modes in MgO nanotubes constructing stacks of squares and hexagons of MgO clusters. Raman spectra of Fe-, Co-, Cr-, and Li-ion-doped MgO were investigated experimentally in [30,31,32,33,34,35].

Let us emphasize that MgO NPs are biocompatible and non-toxic. Therefore, they could be applied in biomedical applications, such as antibacterial/anticancer therapy, magnetic hyperthermia (MHT), nano-cryosurgery, magnetic resonance imaging contrast agent, etc. [36,37,38,39,40]. Boubeta et al. [8] and Chalkidou et al. [37] considered Fe-doped MgO NPs for application in MHT. Boubeta et al. [39] showed that Fe/MgO nanospheres can be used also as a contrast agent in the field of diagnostic magnetic imaging, as well as in drug delivery. Chalkidou et al. [37] studied the heating efficiency of Fe/MgO NPs and their in vitro application in MHT on cancer cells. Ranathunge et al. [41] developed pure MgO nanoflakes (*d* = 20 nm) as drug carriers and loaded them with doxorubicin for use as a targeted drug-delivery system for potential application in cancer therapy. Recently, Almontasser and Parveen [42] studied the effects of Ni, Co and Fe doping concentrations on the antibacterial behaviors of MgO NPs for biomedical applications. The number of oxygen vacancies increases with increasing amounts of dopant ions, leading to an increase in magnetic properties.

In the present paper, we study theoretically the magnetic, optical and phonon properties in ion-doped MgO NPs. To our knowledge, such studies of ion-doped MgO NPs based on microscopic models are lacking. Moreover, we will investigate which MgO NPs are appropriate for MHT.

## 2. The Model and the Method

The spins in our NP are situated in shells numbered n=1,…,N, from the central spin to the surface shell. The distance between the shells is ∼1 nm [23].

MgO crystallizes in the salt structure. The lattice is a face-centered cubic where each oxygen anion has six nearest Mg cationic neighbors and vice versa with a symmetry point group Oh. It is a nonmagnetic insulator with a band gap width of 7.8 eV. The valence zone consists of the oxygen 2p states while the conduction zone is composed of the Mg 3s states [43,44]. The oxides become magnetic after being doped with 3d elements. We consider here MgO doping with Fe${}^{2+}$ (${}^{5}T_{e}$(2$e_{g}4t_{2g}$) and Co${}^{2+}$ (${}^{5}T_{e}$(2$e_{g}5t_{2g}$) ions. With this doping, a red shift is observed at the absorption edge (i.e., the band gap width is reduced). The ions of these transition metals create energy levels in the band gap of MgO, resulting in a change in the band gap width [43,44]. For Fe${}^{2+}$ and Co${}^{2+}$, the $t_{2g}$ states lie below the $e_{g}$ states near the top of the valence zone [43,44]. The 3d localized spins overlap with those of the O-2p orbitals and this is an indication of a strong hybridization between Fe${}^{2+}$ or Co${}^{2+}$ and their nearest-neighbor O atoms, i.e., there is a strong p–d exchange interaction between electrons in the valence zone and localized *d* electrons. The strong p–d Co(Fe)-O hybridization is responsible for the upward shift of the top of the valence zone leading to $E_{g}$ reduction. It must be noted that the face-centered cubic lattice (space group Fm3m) is not changed by Fe or Co ion doping.

The modified Heisenberg model is used to describe the magnetic properties of MgO:

(1) $$\begin{array}{ccc} H_{sp} & = & -\sum_{i,l}xJ_{il}S_{i}.S_{l}-D\sum_{i}{\left(S_{i}^{z}\right)}^{2}\\ & - & \frac{1}{2}\sum_{i,j}F\left(i,j,k\right)Q_{i}S_{j}^{z}S_{k}^{z}-\frac{1}{4}\sum_{i,j,r,s}R\left(i,j,r,s\right)Q_{i}Q_{j}S_{r}^{z}S_{s}^{z}+h.c., \end{array}$$

where $S_{i}$ and $S_{i}^{z}$ are the Heisenberg spin-operators for the localized spins of the doping ion at site *i*, *J* is the nearest-neighbor exchange interaction between the doping ions. *D* is the single-site anisotropy parameter. *h* is an external magnetic field. *x* is the ion-doping concentration. *F* and *R* designate the spin–phonon interaction constants, which are proportional to the first and second derivative of *J*, respectively [45]. $J_{ij}=J\left(r_{i}-r_{j}\right)$ depends on the distance between the spins, i.e., on the lattice parameters, inverse proportional. The surface and doping effects are included using different *J*s on the surface or the doped state and the bulk or undoped ones denoted by the indices “s”, “d” and “b”, respectively.

From the spin Green function $G_{ij}\left(E\right)=\langle \langle S_{i}^{+};S_{j}^{-}\rangle \rangle$ the magnetization $M=\langle S^{z}\rangle$ is observed as:

(2) $$M\left(T\right)=\frac{1}{N}\sum_{i}\left[\left(S+0.5\right)\coth\left[\left(S+0.5\right)\beta E_{mi}\right]-0.5\coth\left(0.5\beta E_{mi}\right)\right],$$

$\beta =1/k_{B}T$, $E_{mi}$ is the spin excitation energy.

$H_{ph}$ contains the lattice vibrations:

(3) $$\begin{array}{ccc} H_{ph} & = & \frac{1}{2!}\sum_{i}\omega_{0i}a_{i}^{+}a_{i}+\frac{1}{3!}\sum_{i,j,r}B\left(i,j,r\right)Q_{i}Q_{j}Q_{r}\\ & + & \frac{1}{4!}\sum_{i,j,r,s}A\left(i,j,r,s\right)Q_{i}Q_{j}Q_{r}Q_{s}. \end{array}$$

$Q_{i}$ and $\omega_{0i}$ are the normal coordinate and frequency of the lattice mode, $a^{+}$ and *a* phonon operators, *B* and *A* anharmonic phonon–phonon interaction constants.

From the phonon Green function

(4) $$G_{ij}\left(t\right)=\langle \langle a_{i}\left(t\right);a_{j}^{+}\rangle \rangle$$

using the method of Tserkovnikov [46] the phonon energy ω and damping γ is calculated, where

(5) $$\omega_{ij}^{2}=\omega_{0}^{2}-2\omega_{0}\left(M_{i}M_{j}R_{ij}\delta_{ij}-\frac{1}{2N}\sum_{r}A_{ijr}^{ph}\left(2{\bar{N}}_{r}+1\right)-B_{ij}^{ph}\langle Q_{ij}\rangle \delta_{ij}\right),$$

with

(6) $$\langle Q_{ij}\rangle =\frac{M_{i}M_{j}F_{ij}\delta_{ij}-\frac{1}{N^{\prime }}\sum_{r}B_{ijr}^{ph}\left(2{\bar{N}}_{r}+1\right)}{\omega_{0}-M_{i}M_{j}R_{ij}\delta_{ij}+\frac{1}{N^{\prime }}\sum_{r}A_{ijr}^{ph}\left(2{\bar{N}}_{r}+1\right)}.$$

The band gap energy $E_{g}$ is defined by the difference between valence and conduction bands:

(7) $$E_{g}=\omega^{+}\left(k=0\right)-\omega^{-}\left(k=k_{\sigma }\right).$$

The electronic energies $\omega^{\pm }\left(k\right)$

(8) $$\omega^{\pm }\left(k\right)=\epsilon_{k}-\frac{\sigma }{2}I\langle S^{z}\rangle +\sum_{k^{\prime }}\left[v\left(o\right)-v\left(k-k^{\prime }\right)\right]\langle n_{k^{\prime }-\sigma }\rangle$$

are observed from Green functions $g\left(k\sigma \right)=\ll c_{k\sigma };c_{k\sigma }^{+}\gg$, σ=±1, $c_{i\sigma }^{+}$ and $c_{i\sigma }$ are Fermi operators using the s(p)–d model [47]. $\epsilon_{k}$ is the conduction band energy calculated in the paramagnetic state, $\langle n_{k^{\prime }\sigma }\rangle$ is the occupation number distribution. *I* is the s(p)–d interaction between spins of the free s(p) electrons and of the localized *d* electrons, and *v* is the Coulomb interaction.

## 3. Numerical Results and Discussion

Bulk MgO is diamagnetic, but Mg${}^{2+}$ is a paramagnetic ion. Here, we will consider how the surface, size and doping effects can change the physical properties of a MgO NP and what the origin is of this change. The following model parameters are used: $J_{b}$ = 75 K, $D_{b}$ = 10 K, *F* = 24 cm${}^{-1}$, *R* = −19 cm${}^{-1}$, *B* = −2.90 cm${}^{-1}$, *A* = 6.60 cm${}^{-1}$.

### 3.1. Size Dependence of Magnetization and Curie Temperature

**Figure 1:**
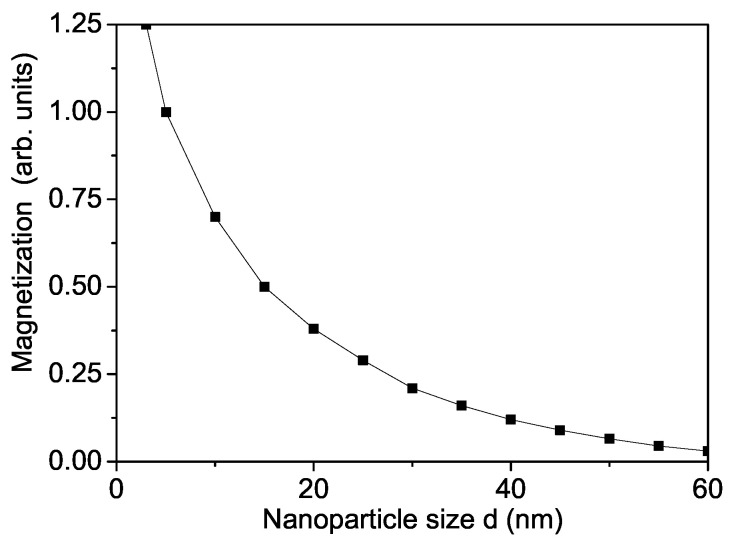
Size dependence of the spontaneous magnetization $M_{s}$ for *T* = 315 K for a pure MgO NP, $J_{s}=1.3J_{b}$.

**Figure 2:**
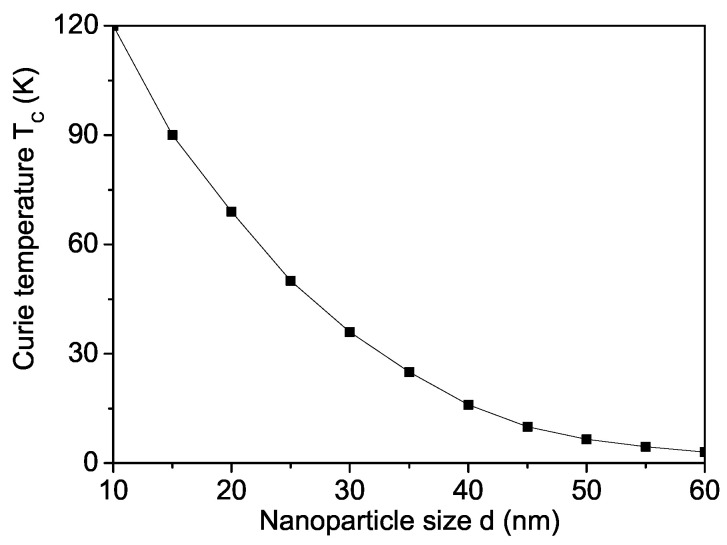
Size dependence of the Curie temperature $T_{C}$ for a pure MgO NP, $J_{s}=1.3J_{b}$.

First, the spontaneous magnetization $M_{s}$ in a pure MgO NP is calculated as a function of the size (Figure 1). Let us emphasize that the variation $M_{s}$ of metal and metal oxide NPs has been a matter of great debate in recent years [48,49]. It can be seen that $M_{s}$ increases with reduced particle size *d* due to uncompensated spins at the surface. Therefore, we choose for *J* on the surface and the bulk the relation $J_{s}>J_{b}$. Our results confirm that there is strong evidence of the existence of intrinsic RTFM in MgO NPs due to cation and Mg vacancies on the surface. The latter can induce local magnetic moments. Unfortunately, there are no reported experimental data for M(d) of MgO NPs. From Figure 2, it can be seen that the Curie temperature $T_{C}$ increases also with decreasing NP size.

### 3.2. Ion-Doping Effects on Magnetization, Curie Temperature and Coercive Field

**Figure 3:**
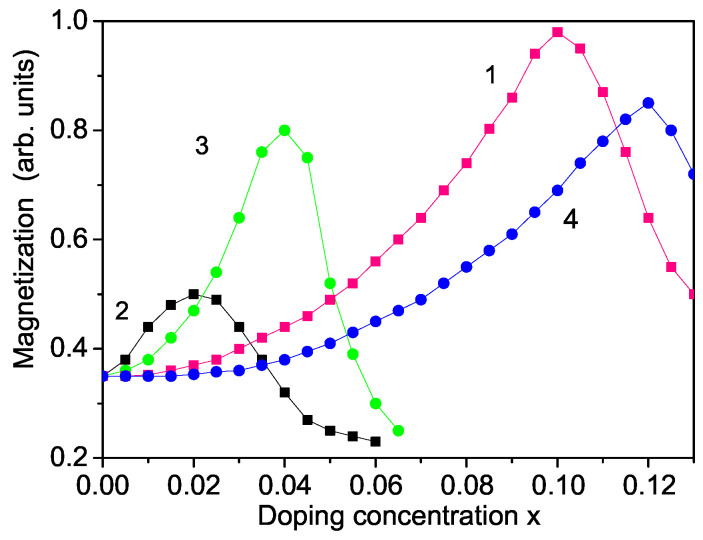
Ion-doping concentration dependence of the magnetization $M_{s}$ for Fe (1), Al (2), La (3) and Co-doped (4) MgO NPs (*T* = 315 K, *d* = 20 nm).

Next, we will consider the doping effects of different doping ions on the spontaneous magnetization $M_{s}$. Obeid et al. [23] observed experimentally that, by Co ion doping, the lattice parameters decrease up to *x* = 0.12. This means that, due to the compressive strain, we must use the relationship $J_{d}>J_{b}$, enhancing the spontaneous magnetization $M_{s}$, as shown in Figure 3, curve 4. Thus, the increase of $M_{s}$ contributes additively to the ferromagnetic coupling between the Co${}^{2+}$ ions (*S* = 3/2) as well as the s–d mechanism.

Mishra et al. [10] reported that, using Al ion doping, the oxygen vacancy concentration has a maximum in dependence on *x* at 0.02. Moreover, the lattice parameters first decrease and then increase. There is a minimum at *x*∼ 0.02. This means that $J_{d}$ would first increase and is larger than $J_{b}$ in the undoped states ($J_{d}>J_{b}$), leading to an increase in spontaneous magnetization $M_{s}$ up to *x* ∼ 0.02, as shown in Figure 3, curve 2. For larger doping concentration, the magnetization $M_{s}$ decreases strongly and is smaller than that for *x* = 0. The nonmonotonic dependence of $M_{s}$ on Al doping concentration could be due to the stabilization of the enhanced oxygen vacancy defects using Al dopants.

Curve 3 in Figure 3 shows the effect of La${}^{3+}$ doping on MgO NP. There is again a maximum in the spontaneous magnetization $M_{s}$ at *x* ∼ 0.04 in agreement with Rani et al. [13] for a La-doped MgO NP. Curve 1 presents the effects of Fe${}^{3+}$ ion (*S* = 5/2) doping on MgO NP. There is a maximum in $M_{s}$ at *x* ∼ 0.1, in coincidence with Borhade et al. [22] (*x* = 0.1) and Phokha et al. [12] (*x* = 0.07). A similar maximum is found also in the spontaneous magnetization $M_{s}$ in Pb and Sc doped MgO NP, at *x* = 0.03 and 0.15, respectively, by Najem et al. [11]. Let us emphasize that the spin–phonon interaction, which is important in MgO [50,51] and is included in our model, renormalizes *J* to $J^{eff}=J+2F^{2}/\left(\omega_{0}-MR\right)$ and leads to enhanced $M_{s}$ and $T_{C}$ in MgO NPs.

It must be mentioned that at low doping concentration *x* plays a major role in the magnetic ordering of the long-acting ferromagnetic s(p)–d interaction. The magnetic moments will be arranged collinearly, and the net magnetic moment will increase. With increasing *x* of magnetic ions, the short-acting exchange interaction *J* starts to compete with the s(p)–d interaction, and to oppose the collinear arrangement of spins. This is the reason a maximum appears in the $M_{s}$ curve at a finite value of *x*. Above this value, *J* begins to prevail over the s(p)-d interaction, which leads to a decrease of the magnetization $M_{s}$ with increasing *x*.

**Figure 4:**
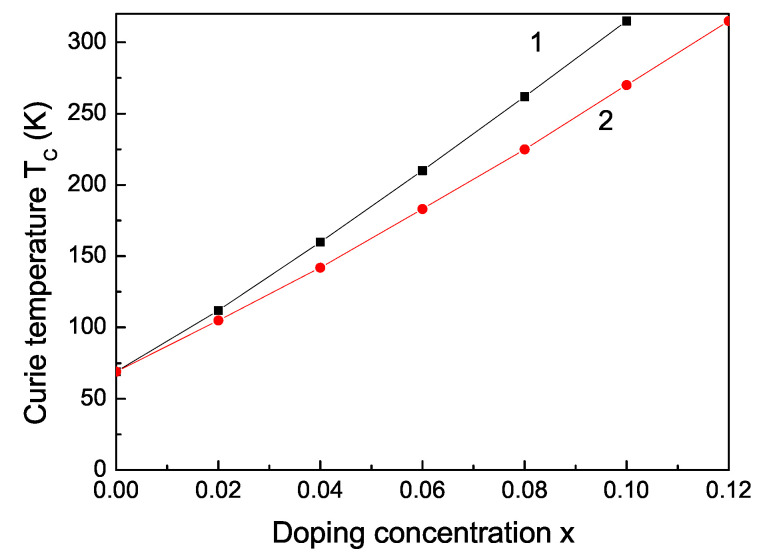
Ion-doping concentration dependence of the Curie temperature $T_{C}$ of a MgO NP, *d* = 20 nm, for Fe (1), Co (2).

Figure 4 shows the increase of the Curie temperature $T_{C}$ of a MgO NP, *d* = 20 nm, as a function of the Fe and Co concentration *x*. The $T_{C}$ increase could be due to the p–d exchange coupling between doping and host ions. Moreover, using the substitution, the compressive deformations lead to a decrease of the distances between the localized spins and an increase of the super-exchange interaction constant *J* between them, which naturally leads to an increase of $T_{C}$. It must be noted that $T_{C}$ has also a maximum, because FeO and CoO are antiferromagnetic with a Neel temperature of $T_{N}$ = 198 K and 291 K, respectively, (not considered here) [52].

**Figure 5:**
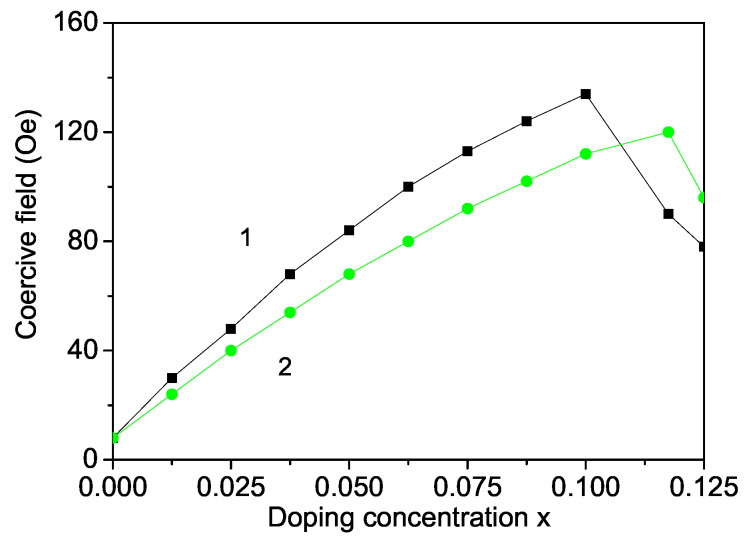
Doping concentration dependence of the coercive field of a MgO NP for *d* = 20 nm, *T* = 315 K for different doping ions: (1) Fe and (2) Co ion doping.

A maximum in the coercive field $H_{c}$ is obtained as a function of the Fe and Co ion dopants (see Figure 5). Bures et al. [53] also reported an increase of $H_{c}$ in Fe-doped MgO NPs for x<0.1. Unfortunately, above this value there are no experimental data. Figure 5 clearly shows that the Co and Fe-doped MgO NPs become more magnetic. The increase in the concentration of 3d ions with their strong single-ion magnetic anisotropy leads to an increase in the coercive field. This increase is also a consequence of surface effects by which the surface magnetic anisotropy constant is usually at least one order of magnitude greater than that of the bulk.

In summary, the RTFM and the magnetic phase transition temperature in these materials is due to a competition between a long-acting, free-charge-assisted exchange interaction between magnetic impurities, which is responsible for the ferromagnetic ordering of local magnetic moments, and a short-acting super-exchange interaction between the magnetic ions of impurity transition metals. This competition depends strongly on the *x* concentration of the impurity ions, which is expressed in the appearance of a maximum in the magnetization curve as a function of *x*. Let us emphasize that the numerical calculations presented so far in paragraphs 3.1 and 3.2 are in good qualitative agreement with the cited experimental results, and are evidence of the adequacy of our proposed microscopic model.

### 3.3. Application of Fe-Doped MgO NPs for MHT

**Figure 6:**
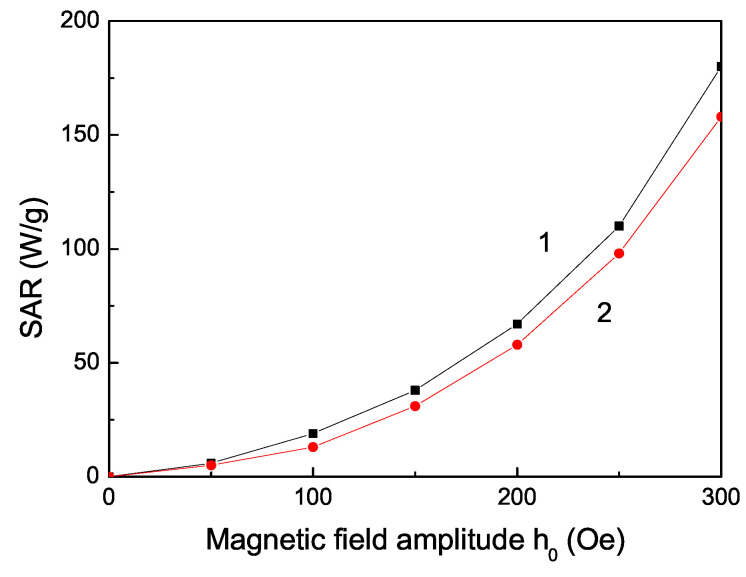
Dependence of SAR on the magnetic field amplitude $h_{0}$ of a Fe (*x* = 0.1) (1) and Co (*x* = 0.12) (2) doped MgO NP, *d* = 20 nm, for *f* = 100 kHz and *T* = 315 K.

Magnetic hyperthermia (MHT) is the most significant cancer therapy form using magnetic nanoparticles [54,55]. Therefore, we will now search for some ion-doped MgO NPs that are appropriate for MHT. The conditions for these magnetic NPs for in vitro and in vivo treatment of tumors are: (i) a Curie temperature around 42 °C (315 K); (ii) a large saturation magnetization $M_{s}$; (iii) large value of the coercive field $H_{c}$; (iv) high specific (heat) absorption rate value; (v) size of NPs less than 30 nm; and (vi) bio-compatibility with human cells. The MgO NPs are biocompatible and non-toxic [56], as are the Fe and Co ions, too. Let us emphasize that MgO shows weak ferromagnetism but doping with some suitable dopants can enhance the magnetism and hyperthermia efficiency. The Fe- and Co-doped MgO NPs have a large spontaneous magnetization $M_{s}$ and a large coercive field $H_{c}$ (see Figure 3 and Figure 5). It can be seen from Figure 4 that the Fe- and Co-doped MgO NPs with a size of 20 nm and Fe- and Co-doping concentration *x* = 0.1 and *x* = 0.12, respectively, have a Curie temperature $T_{C}$ = 315 K. The MHT is based on the simple physical fact that when magnetic NPs are subjected to an alternating magnetic field, they produce heat. The heating ability of magnetic NPs is expressed by the specific absorption rate (SAR). Generally, the SAR values depend on external parameters (amplitude of magnetic field amplitude $h_{0}$ and its frequency *f*) and internal parameters: magnetic NP structure (size, shape, crystal structure and doping); magnetic properties (magnetic anisotropy, magnetization, coercivity). SAR is calculated for a Fe- and Co-doped MgO NP, *d* = 20 nm, *T* = 315 K (see Figure 6). The SAR effect raises with increasing magnetic field amplitude $h_{0}$ in good agreement with [37] for Fe-doped MgO NPs. This raising of SAR is because the area of the hysteresis curve increases, whose area is proportional to the hysteresis losses and therefore to the increase in the heat transfer to the tumor. SAR is a quantitative feature of this transfer. We observe a quadratic SAR dependence on the amplitude of the applied magnetic field in accordance with the analytical expression reported in [57]: $SAR∼fh_{0}^{2}M_{s}^{2}V/k_{B}T$. All these results show that these NPs are appropriate for application in MHT. Let us emphasize that Boubeta et al. [8] and Chalkidou et al. [37] also considered Fe-doped MgO NPs for application in MHT. Ranathunge et al. [41] investigated pure MgO NPs (*d* = 20 nm) for application in cancer therapy. Recently, Almontasser and Parveen [42] studied Ni, Co and Fe-doped MgO NPs for biomedical applications.

### 3.4. Size and Doping Effects on Band Gap Energy

**Figure 7:**
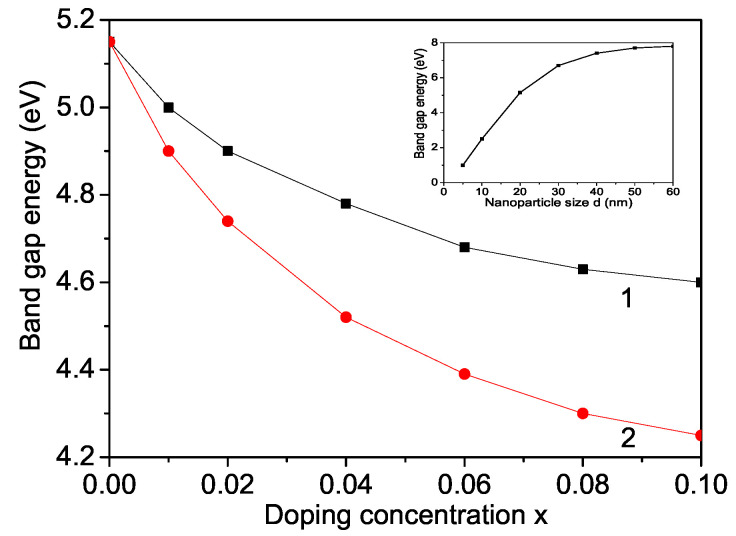
Ion-doping concentration dependence of the band gap energy for a (1) Co and (2) Fe-ion-doped MgO NP for $J_{d}=1.3J_{b}$. Inset: $E_{g}\left(d\right)$ for pure MgO.

Next, we will study the band gap energy $E_{g}$ in MgO NPs as a function of size and ion doping. MgO is a wide band gap oxide ($E_{g}$ = 7.8 eV) and, unlike semiconductors, requires the use of VUV radiation sources (synchrotron radiation) [58,59]. In pure MgO, the maximum valence band near the Fermi level mainly contributes to the $O_{2P}$ state, while in the conduction band there is a small appearance of $Mg_{2P}$ and $Mg_{3S}$ states, which indicates the ionic nature of the bonds between Mg and O atoms [60]. The band gap of a pure MgO NP is smaller than that of the bulk one, e.g., for *d* = 20 nm it is calculated to be 5.15 eV (see inset in Figure 7). A similar decrease of $E_{g}$ in MgO nanostructures is observed in [18,61,62,63,64,65,66]. The results for Co or Fe ion-doped MgO NPs are presented in Figure 7, curves 1 and 2. $E_{g}$ is reduced with increasing Co or Fe dopants due to the compressive strain, in agreement with Obeid et al. [23], Borhade et al. [22], Raza et al. [67]. It must be noted that Almontasser et al. [42] observed, in disagreement with us, an increase of $E_{g}$ with increasing Fe and Co dopants. As already mentioned, the occupied defect levels of Fe- and Co-doped MgO are above the top of the valence band [43] and increase with increasing Fe dopants. They move towards the bottom of the conduction band. The presented numerical results confirm that the reduction of the band gap width with increasing *x* (for *d* = const) is due to the strong hybridization of the 3d states of the transition metal ions with the 2p states of the host oxygen matrix. Since the energies of the *p*-orbitals of the oxygen anions are very close to those of the Co (Fe) *d*-states, the strong oxygen–transition metal exchange interaction has a determining role in the reducing of $E_{g}$. The ionic radii of Co${}^{2+}$ (0.065 nm) and Fe${}^{2+}$ (0.064 nm) are smaller than that of Mg${}^{2+}$ (0.072 nm), i.e., the lattice parameters decrease ($J_{d}>J_{b}$). Therefore, we observe an enhanced magnetization for small doping concentration and from Equation (6), for the electronic energies, it can be seen that this leads to smaller electronic energies and following also to smaller $E_{g}$. This coincides with the result of Lopez et al. [68]. The authors have investigated $E_{g}$ in CdS thin films.

Our model would also explain the decrease of the band gap $E_{g}$ when raising Co, Ca, Li, Ce or Ag dopants, which has been found experimentally by [23,69,70]. Therefore, the doped MgO NPs could find application in photocatalysis [71,72].

As can be seen from Equations (5) and (6), the width of band gap $E_{g}$ depends on the competition between the exchange interaction constant *J*, the s(p)–d and the Coulomb *v* interactions. Strong s(p)–d interaction leads to a decrease, whereas the Coulomb interaction *v* leads to an enhancement of $E_{g}$.

### 3.5. Size and Doping Effects on the Phonon Spectrum

**Figure 8:**
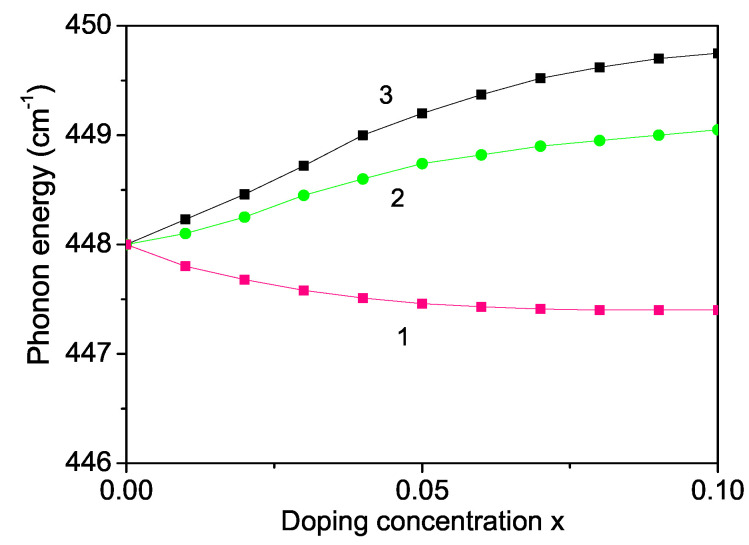
Ion-doping concentration dependence of the phonon mode ω = 448 cm${}^{-1}$ of a MgO NP, *d* = 20 nm, for *T* = 20 K and different ions: (1) Sr with $R_{d}=0.8R_{b}$; (2) Fe, $R_{d}=1.2R_{b}$; (3) Co, $R_{d}=1.4R_{b}$.

Finally, we will calculate the phonon spectrum as a function of size and ion doping. It is supposed that in MgO NPs a 448 cm${}^{-1}$ line is associated with a transverse optical phonon [29,73]. The phonon energy increases with decreasing NP size (not shown here) in agreement with [73,74]. It must be noted that the surface phonons are distinct from the bulk ones, as they arise from the abrupt termination of a crystal structure at the surface of a solid. As discussed in Section 3.1. the surface effects lead to the relationship between the exchange interaction constants $J_{s}>J_{b}$ and therefore we have for the spin–phonon constants the relationship $|R_{s}|>|R_{b}|$, which is the origin for the increase of the phonon energy with decreasing NP size. We have investigated the ion-doping dependence of the phonon mode ω = 448 cm${}^{-1}$ for different doping ions. The ionic radius of Mg${}^{2+}$ (0.72 $\dot{A}$) is smaller compared to that of Ca${}^{2+}$ (1 $\dot{A}$), Ba${}^{2+}$ (1.35 $\dot{A}$), Sr${}^{2+}$ (1.18 $\dot{A}$) or Y${}^{3+}$ (0.9 $\dot{A}$) [75], i.e., the interaction constants are $J_{d}<J_{b}$ and $R_{d}<R_{b}$. Therefore, we obtain a decrease of the phonon energy with increasing Sr doping concentration; see Figure 7, curve 1. Similar behavior could also be observed for a Ca, Ba or Y-doped MgO NPs. The radius of doped Co${}^{2+}$ (0.685 $\dot{A}$) or Fe${}^{3+}$ (0.69 $\dot{A}$) ions is smaller compared to that of the Mg host ion (0.72 $\dot{A}$), i.e., we have a compressive strain with $J_{d}>J_{b}$ and $R_{d}>R_{b}$. The phonon energy ω for Fe or Co-doped MgO NPs raises when increasing the ion dopants. The results are presented in Figure 8, curves 2, 3, respectively.

**Figure 9:**
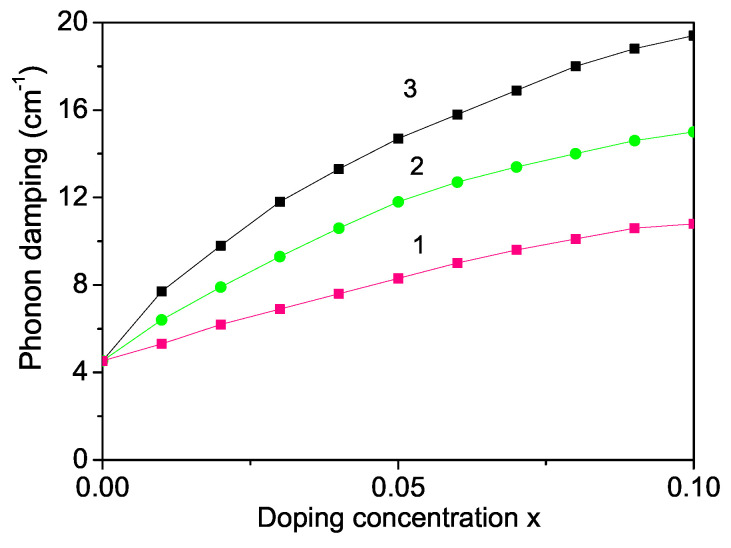
Ion-doping concentration dependence of the phonon damping γ of a MgO NP, *d* = 20 nm, for *T* = 20 K and different ions: (1) Sr with $R_{d}=0.8R_{b}$; (2) Fe, $R_{d}=1.2R_{b}$; (3) Co, $R_{d}=1.4R_{b}$.

The phonon damping corresponding to the full width of the half maximum increases with increasing the doping concentration for all doping ions, i.e., the Raman peaks become wider (see Figure 9). Unfortunately, there are no experimental data for the doping concentration of the phonon modes in MgO NPs. It must be noted that the electron–phonon interaction is important for the surface lattice phonons in MgO NPs, as shown recently by Sibaja et al. [76], which will be considered in a future paper.

## 4. Conclusions

In conclusion, using a microscopic model has shown that spontaneous magnetization $M_{s}$ increases with decreasing NP size due to surface and size effects. To clarify the mechanism responsible for the experimentally observed RTFM in ion-doped MgO NPs, Co, Fe, Al and La-doped MgO NPs are considered. We have shown that there is a connection between microstructure and macroscopic magnetic behavior. $M_{s}$ shows a maximum value in dependence on the Co, Fe-, Al- and La-doping concentration *x*. A RTFM is due to the interaction between the doped and host ions. Moreover, enhanced ferromagnetic properties are observed for small ion-doping concentration. Our next goal is to find such magnetic NPs that can be applied for MHT and cancer therapy. It is shown that Fe and Co-doped MgO NP for *x* = 0.1 and *x* = 0.12, respectively, with size of around 20 nm, has a Curie temperature $T_{C}$ = 315 K, with increasing SAR values when increasing the magnetic field amplitude, i.e., they are appropriate for application in MHT. Band gap energy $E_{g}$ is reduced with increasing Co and Fe ion concentration. Phonon energy increases with decreasing NP size. The phonon mode ω = 448 cm${}^{-1}$ increases or decreases with increasing Co and Fe, or Sr ion-doping concentration, respectively, whereas phonon damping increases with concentration for all doping ions.

## Data Availability

The raw data that support the findings of this study are available from the corresponding author upon reasonable request.
